# Intraoperative irrigation of artificial cerebrospinal fluid and temperature of irrigation fluid for chronic subdural hematoma: a systematic review and meta-analysis

**DOI:** 10.3389/fneur.2023.1218334

**Published:** 2023-07-06

**Authors:** Yong-Wei Huang, Zong-Ping Li, Xiao-Shuang Yin

**Affiliations:** ^1^Department of Neurosurgery, Mianyang Central Hospital, School of Medicine, University of Electronic Science and Technology of China, Mianyang, Sichuan, China; ^2^Department of Immunology, Mianyang Central Hospital, School of Medicine, University of Electronic Science and Technology of China, Mianyang, Sichuan, China

**Keywords:** chronic subdural hematoma, irrigation fluid, temperature, recurrence, meta-analysis

## Abstract

**Purpose:**

To systematically review the different types of irrigation fluid and the different temperatures of irrigation fluid on postoperative recurrence rates in the evacuation of chronic subdural hematoma (CSDH).

**Methods:**

We conducted a comprehensive search of electronic databases, including PubMed, Embase, the Cochrane Library, the China National Knowledge Infrastructure (CNKI), WanFang, the Chinese VIP Information (VIP), and China Biology Medicine (CBM), and reference lists of relevant studies to identify all eligible studies. Two reviewers independently screened the titles and abstracts for inclusion, and the full-text articles were assessed for eligibility based on predetermined inclusion and exclusion criteria. Data were extracted using a standardized form, and the quality of the studies was assessed using a risk of bias tool. Meta-analyses were performed using a fixed-or random-effects model, and heterogeneity was assessed using the I2 statistic. The primary endpoint was the postoperative recurrence rate.

**Results:**

After stringent screening, a total of 11 studies were identified, including six English publications, four Chinese publications, and one Japanese publication, involving a population of 29,846 patients. Our meta-analysis provides evidence that artificial cerebrospinal fluid (ACF) could decrease the post-operative recurrence rate by 47% after the evacuation of CSDH when compared to normal saline (NS) [(odds ratio) OR 0.53, 95% confidence intervals (CI): 0.31–0.90, *p* = 0.02, *I*^2^ = 67%]. Besides, the irrigation fluid at body temperature could decrease the postoperative recurrence rate of CSDH by 64% when compared to room temperature (OR = 0.36, 95% CI = 0.22–0.59, *p* < 0.0001, *I*^2^ = 0%).

**Conclusion:**

Our analysis revealed significant difference in the choice of irrigation fluid for CSDH surgery. Notably, we found that irrigation with fluid at body temperature demonstrated superiority over irrigation with fluid at room temperature, resulting in fewer instances of recurrence. This straightforward technique is both safe and widely available, providing an opportunity to optimize outcomes for patients with CSDH. Our findings suggest that the use of body-temperature NS or ACF of room temperature during operation should be considered a standard of procedure in CSDH surgery. Nevertheless, whether the different temperature of ACF could be considered a standard of procedure in CSDH surgery still need high-quality RCTs to further identify.

**Systematic review registration:**

https://www.crd.york.ac.uk/prospero/; Identifier CRD42023424344.

## Introduction

Chronic subdural hematoma (CSDH) is a condition characterized by the accumulation of aged and degraded blood products within the subdural space of the brain. This process is driven by inflammatory and angiogenetic factors, which can lead to a significant increase in volume and subsequent compression of brain tissue, resulting in a range of symptoms (1). Common symptoms of CSDH include focal neurological deficits, altered mental states, and signs of increased intracranial pressure such as headaches, decreased consciousness, and, in severe cases, death. Older adults, particularly those aged over 65 years, are at heightened risk of developing CSDH due to factors such as brain atrophy, increased frequency of anticoagulant therapy, and a higher incidence of head trauma caused by falls (2). The growing incidence of CSDH is largely attributed to an aging population (3), reaching 58 patients per 100,000 people in those aged 65 years old or more (4). Data from the Nordic countries indicate that the incidence of CSDH has almost tripled over the past 20–30 years, with a corresponding doubling of surgical procedures (5, 6). As one of the most populous countries in the world, China has many elderly people. Therefore, the prevention and treatment of CSDH are particularly important.

The most frequent surgical approach for CSDH is evacuation through one or two burr holes, followed by the insertion of a postoperative drain (7). During surgery, subdural space irrigation is commonly performed (4, 8). However, despite these measures, recurrence rates of CSDH requiring reoperation remain at approximately 10 to 20% (9). Such recurrences are associated with a substantial increase in morbidity and mortality (10, 11). Therefore, reducing recurrence rates is of great importance, not only to improve outcomes for individual patients but also to optimize healthcare resource utilization.

Artificial cerebrospinal fluid (ACF) is an irrigating solution that mimics the composition of human cerebrospinal fluid (CSF). Its efficacy has been demonstrated by over 1,000 medical facilities in Japan where ACF has been commercially available (12). ACF has been shown to possess hemostatic properties and to reduce cerebrovascular permeability, making it a promising irrigation solution for patients with CSDH undergoing burr-hole surgery (13, 14). The use of ACF during surgery may lead to improved outcomes for these patients. Comparative studies have been conducted in recent years to investigate the efficacy of normal saline (NS) and ACF as irrigation solutions in CSDH burr hole surgery (12, 15–17). These retrospective single-center studies have suggested that irrigation with ACF may reduce the recurrence rate of CSDH. However, there is a dearth of multi-center, prospective, randomized controlled trials in this field. Moreover, there has been limited research on the impact of irrigation solutions on postoperative complications in patients undergoing CSDH surgery. Future research should aim to address these gaps in knowledge.

The temperature of the irrigation fluid used during surgery for CSDH may impact recurrence rates, as higher solubility with body temperature fluid may aid in CSDH clearance and lower the risk of coagulation issues (18). Despite these potential benefits, it remains common practice to use irrigation fluid at room temperature (19). A poll conducted in 2017 among 620 neurosurgeons found that 57% used body temperature irrigation, 40% used room temperature, and 3% did not use irrigation at all (20). In order to examine the influence of irrigation fluid temperature on the rates of recurrence in CSDH patients, Bartley et al. (21) conducted a preliminary investigation. Their findings revealed that adjusting the irrigation fluid temperature to match the body temperature resulted in a reduction of recurrences requiring reoperation from 13.4 to 4.5%. To date, the results of investigating the impact of the irrigation fluid temperature and different irrigation fluids on recurrence rates are limited. And the findings are controversial. Hence, we conducted a systematic review and meta-analysis to assess the efficacy of different irrigation fluids and irrigation fluid temperatures in influencing the postoperative recurrence rate.

## Methods

### Search strategy

This systematic review and meta-analysis adhered to the Preferred Reporting Items for Systematic Reviews and Meta-Analyses (PRISMA) guidelines (22) and was prospectively registered with PROSPERO^1^ under the identifier CRD42023424344 (23). The PRISMA checklist can be found in Supplementary Table S1. Our comprehensive search encompassed multiple databases including PubMed, Embase, the Cochrane Library, Scopus, and the Web of Science, employing the following search terms: (“chronic subdural hematoma” or “CSDH”) AND (“irrigation” or “fluids” or “artificial cerebrospinal fluid” or “ACF” or “normal saline” or “NS” or “temperature”). Additionally, Chinese databases such as the China National Knowledge Infrastructure (CNKI), WanFang, Chinese VIP Information (VIP), and China Biology Medicine (CBM) were searched using the terms: (“慢性硬膜下血肿” AND “冲洗”). To ensure inclusiveness, we also searched ClinicalTrials.gov and WHO-ICTRP. The search was conducted on May 2, 2023. Language restrictions were not applied, and all relevant articles were considered for inclusion. The detailed search strategy is outlined in Supplementary Table S2.

### Study selections

We included studies that met the following PICO (Population, Intervention, Comparisons, and Outcomes) criteria: (1) Population: CSDH patients, including unilateral or bilateral; (2) Intervention: burr-hole irrigation, burr-hole drainage, or YL-1 type hard-channel drilling drainage; (3) Comparisons: ACF vs. normal saline (NS), and body temperature vs. room temperature; (4) Outcomes: post-operative recurrence rate. We excluded systematic reviews and meta-analyses, case reports, reviews, commentaries, and conference abstracts.

Two reviewers (Y-WH and X-SY) independently screened the titles and abstracts of all retrieved records. The same two reviewers separately reviewed the relevant studies in full text and either included or excluded articles based on the eligibility criteria. In cases of discordance, the corresponding authors (Z-PL) made the final decision.

### Data extraction

Two reviewers (Y-WH and X-SY) independently extracted data into separate Excel spreadsheets. The spreadsheets were then cross-checked against each other and the source material to ensure accuracy. We collected the following data: first author name, publication year, country, study design, sample size, age, sex, type of CSDH, type of surgery, primary outcomes, and follow-up duration. If we found any discrepancies, we resolved them by consulting the corresponding authors (Z-PL).

### Study outcomes

The primary outcome of this study was post-operative recurrence, defined as reoperation of the same side within 6 months after the first surgery.

### Bias assessment

Two independent reviewers (Y-WH and X-SY) evaluated the risk of bias in the included studies using the Newcastle-Ottawa Scale (NOS) tool (24) for non-randomized controlled trials (RCTs) and the Cochrane Collaboration’s tool (25) for RCTs in a blinded manner. To ensure accuracy, the risk of bias summaries were cross-checked, and any unresolved discrepancies were resolved by the corresponding authors (Z-PL).

### Statistical analysis

For binary outcomes, we calculated odds ratios (ORs) and their corresponding 95% confidence intervals (CIs). Mean and standard deviation (SD) were estimated using sample size, median, and interquartile range, employing optional estimating methods from McGrath et al. (26), which can be accessed at https://smcgrath.shinyapps.io/estmeansd/. To address clinical heterogeneity, we conducted meta-analyses and subgroup analyses using either random-effects or fixed-effects models (27). Heterogeneity was assessed using the Cochrane Q test, considering *p* < 0.1 or *I*^2^ > 50% as indicators of significant heterogeneity (28). Statistical significance was defined as *p* < 0.05. Funnel plots were utilized to assess publication bias. All statistical analyses were performed using Review Manager software (version 5.3.3; https://training.cochrane.org/online-learning/core-software-cochrane-reviews/revman).

## Results

### Study selection

**Figure 1:**
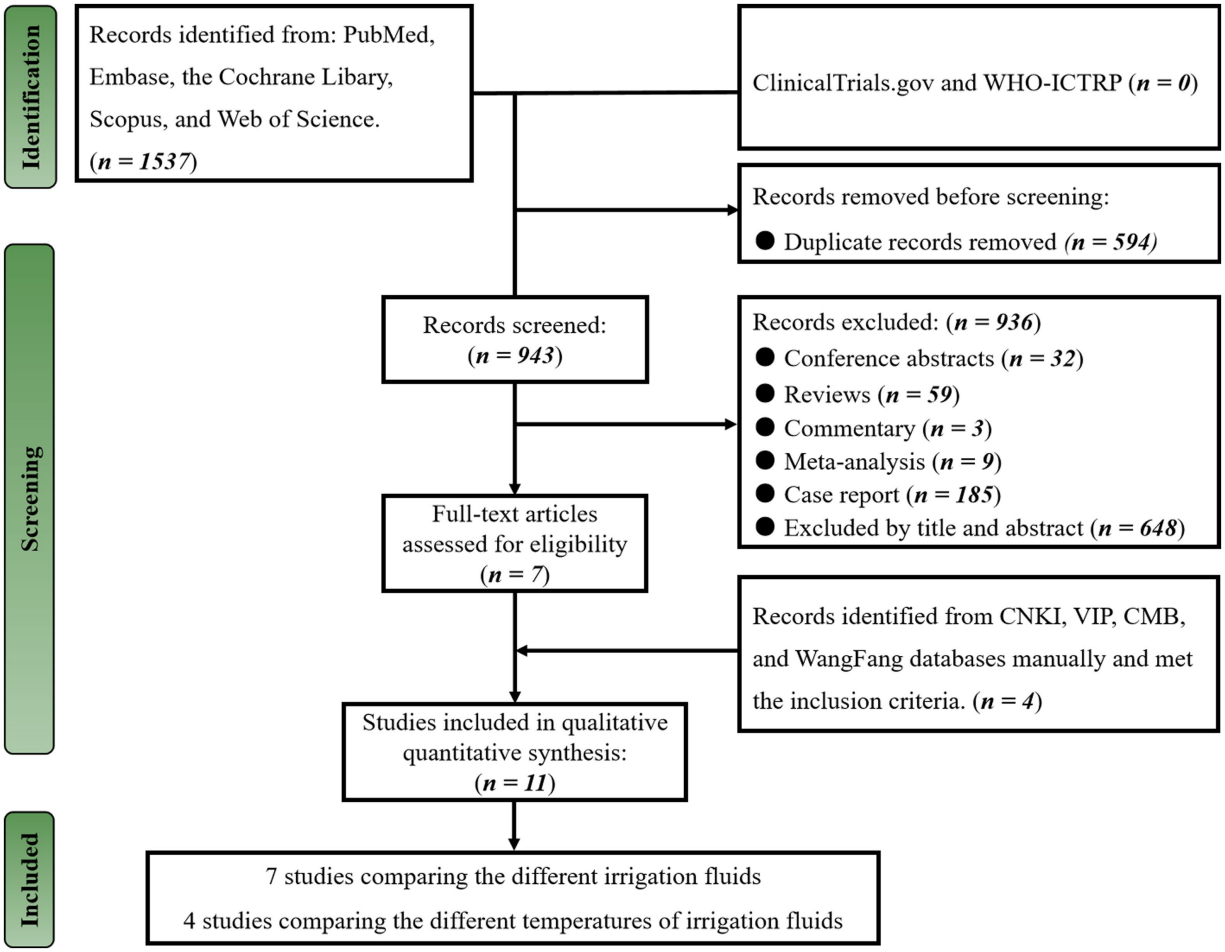
PRISMA flowchart of included studies.

On May 2, 2023, a total of 1,537 publications were identified through the search strategy in English databases. After removing 594 duplicates, we screened the remaining 943 publications based on article type, title, and abstracts and excluded 936 unrelated publications, leaving seven studies. We meticulously evaluated the seven publications for potential eligibility (12, 15, 16, 21, 29–31), and all met inclusion criteria. Subsequently, we manually searched the Chinese database for relevant studies and found four additional studies (32–35) that met our inclusion criteria. In summary, we included a total of 11 studies (12, 15, 16, 21, 29–35) in our systematic review and meta-analysis (Figure 1).

### Characteristics of the included studies

The 11 studies included in this systematic review and meta-analysis were published between 2012 and 2023. Among them, four articles were RCT (30, 31, 33, 34), and the remaining seven articles (12, 15, 16, 21, 29, 32, 35) were non-RCTs. The studies were conducted in China (*n* = 4), Sweden (*n* = 2), and Japan (*n* = 5), with a total of 29,846 patients included. One study (29) utilized a 1:1 propensity score matching (PSM) method to balance the influence of potential confounding factors, resulting in the inclusion of 27,788 patients in the analysis. 10 studies (12, 15, 16, 21, 29–34) underwent burr-hole surgery with drainage or irrigation. One study (35) underwent YL-1-type hard-channel drilling drainage. The follow-up period ranged from 3 months to 1-year after discharge. The results of the included studies are summarized in Table 1.

**Table 1:** The baseline characteristics of included studies.

Author	Year	Nation	Study design	Participants (*n*)	Male (%)	Age (*y*)	Research topic	Type of CSDH	Type of surgery	Primary outcomes	Follow-up	NOS
Adachi et al.(12)	2014	Japan	non-RCT	120	63.3	76.75 ± 11.43	ACF vs. NS	Unilateral	prefrontal burr-hole and overnight drainage	Recurrence	6 months	7
Kuwabara et al.(15)	2017	Japan	non-RCT	234	67.1	64.07 ± 14.26	ACF vs. NS	Unilateral or bilateral	Burr-hole irrigation	Recurrence	3 months	6
Shibahashi et al.(29)	2023	Japan	non-RCT	27,788 in PSM	68.9	40–90	ACF vs. NS	Unilateral or bilateral or not specified	Burr-hole	Recurrence	1 year	8
Wang et al.(33)	2014	China	RCT	160	54.4	36–79	ACF vs. NS	Unilateral	Burr-hole	Recurrence	6 months	※
Wang et al.(34)	2012	China	RCT	80	58.8	36–79	ACF vs. NS	Unilateral	Burr-hole	Recurrence	6 months	※
Takayama et al.(16)	2012	Japan	non-RCT	239	66.5	16–94	ACF vs. NS	Unilateral or bilateral	Burr-hole	Recurrence	3 months	6
Toi et al.(31)	2019	Japan	RCT	342	72.5	75.41 ± 2.84	ACF vs. NS	Unilateral or bilateral	Burr hole drainage	Recurrence	3 months	※
Bartley et al.(21)	2020	Sweden	non-RCT	172	43.8	74.79 ± 12.47	NS (BT) vs. NS (RT)	Unilateral or bilateral	Burr hole	Recurrence	6 months	7
Bartley et al.(30)	2022	Sweden	RCT	541	73.0	75.80 ± 9.80	NS (BT) vs. NS (RT)	Unilateral or bilateral	Burr hole	Recurrence	6 months	※
Huang et al.(32)	2021	China	non-RCT	84	38.6	68.25 ± 8.27	NS (BT) vs. NS (RT)	Unilateral	Burr hole	Recurrence	6 months	7
Zhang et al.(35)	2021	China	non-RCT	86	74.4	66.53 ± 7.12	NS (BT) vs. NS (RT)	Unilateral or bilateral	YL-1-type hard-channel drilling drainage	Recurrence	6 months	6

※ The Cochrane collaboration’s tool was used for RCTs.

CSDH, chronic subdural hematoma; PSM, propensity score matching; ACF, artificial cerebrospinal fluid; NS: normal saline; BT: body temperature; RT, room temperature; NOS, Newcastle-Ottawa Scale; RCT, randomized controlled trials.

### Effect of ACF on recurrence

**Figure 2:**
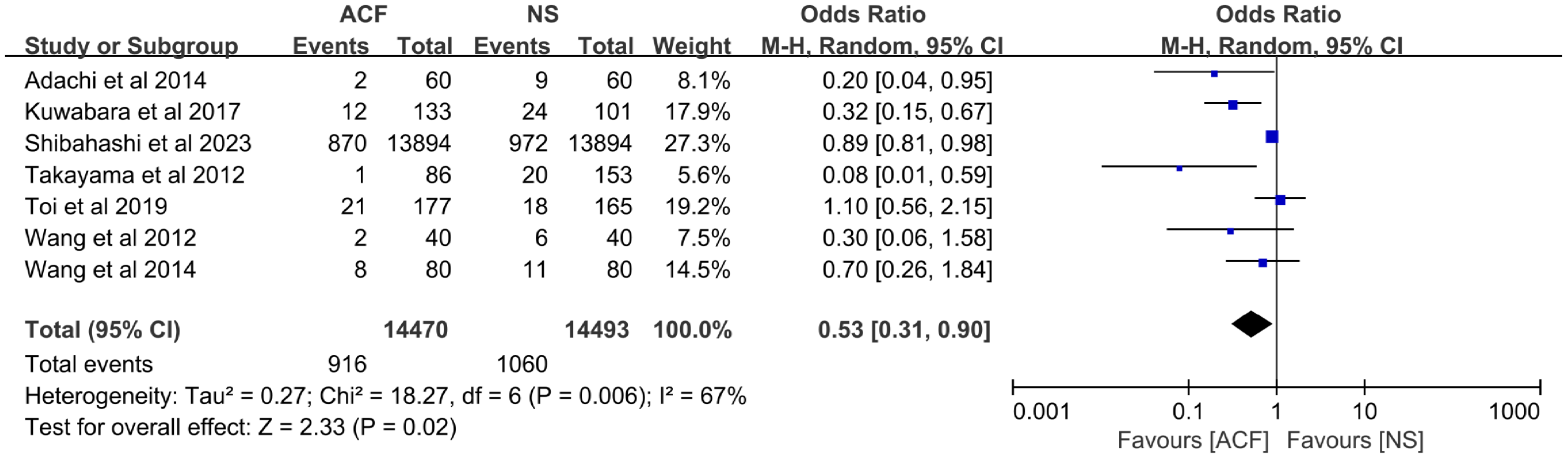
Meta-analysis of postoperative recurrence rates between the ACF group and the NS group.

Seven studies (12, 15, 16, 29, 31, 33, 34) looked at the effect of different irrigation fluids on postoperative recurrence, including 28,963 patients. Among them, 14,470 cases were in the ACF group with 916 patients of recurrence and 14,493 cases in the NS group with 1,060 patients of recurrence. Meta-analysis showed some heterogeneity with I^2^ = 67%, so a random-effects model was used for the analysis, which showed that the CSDH recurrence rate was lower in the ACF group than in the NS group (OR = 0.53, 95% CI = 0.31–0.90, *p* = 0.02) (Figure 2). In other words, the CSDH recurrence rate in the ACF group was decreased by 47% than that in the NS group.

### Effect of the temperature of irrigation fluid on recurrence

**Figure 3:**
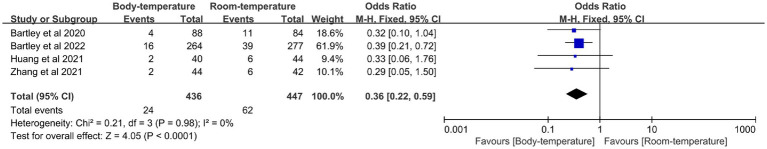
Meta-analysis of postoperative recurrence rates between the body-temperature group and the room-temperature group.

Four studies (21, 30, 32, 35) focused on the different temperatures of irrigation fluids in influencing postoperative recurrence, including 883 patients. Among them, 436 cases were in the body temperature group with 24 patients of recurrence and 447 cases in the room temperature group with 62 patients of recurrence. Meta-analysis showed no heterogeneity with I^2^ = 0%, so a fixed-effects model was used for the analysis, which showed that the CSDH recurrence rate was lower in the body temperature group than in the room temperature group (OR = 0.36, 95% CI = 0.22–0.59, *p* < 0.0001; Figure 3). In other words, the CSDH recurrence rate in the body temperature group was decreased by 64% than that in the room temperature group.

### Risk of bias assessment and publication bias assessment

The NOS has assessed and awarded a median of seven stars to seven studies, with an range of 6 to 8 stars. Cochrane Collaboration’s tool was used to assess RCTs. The methodological quality of the studies included can be found in Supplementary Table S3 and Supplementary Figure S1. Additionally, the probability of publication bias was evaluated through funnel plot results, which are displayed in Supplementary Figure S2.

## Discussion

Chronic subdural hematoma (CSDH) can cause various neurological deficits and is an important risk factor affecting the quality of life of the elderly. Burr hole drainage is a common treatment for CSDH and is considered the preferred method due to its simplicity, short surgery time, minimal trauma, and high safety for first-time CSDH cases. However, due to various factors such as advanced age, brain atrophy, use of anticoagulants, liver and kidney dysfunction, and the formation of intrahematomal septa, postoperative hematoma recurrence is common in CSDH patients. Therefore, adequate intraoperative irrigation and postoperative drainage are crucial. Some studies demonstrated that irrigation with ACF could decrease the rate of CSDH recurrence (12, 15, 16, 29). Nevertheless, some other studies showed that no differences in recurrence rate were seen between ACF and NS (31, 33, 34), and ACF offers sufficient safety as an irrigation fluid for CSDH (31). Wang et al. (33) think that adequate intraoperative irrigation and adequate postoperative drainage are essential to reduce postoperative recurrence rates, and irrigation fluid is not a decisive factor.

In recent years, with the popularity of neuroendoscopic surgery, ACF has been increasingly used in the treatment of neurosurgical diseases. In intracranial aneurysm clipping, ACF is used for intracerebral pool flushing to prevent postoperative vasospasm; lateral ventricular puncture followed by ACF replacement with lumbar pool drainage for severe infections such as meningitis; and ACF replacement for hypertensive ventricular hemorrhage, all of which have achieved better efficacy compared with traditional therapies. ACF is formulated with reference to the concentration of the main biochemical substances in normal CSF, and its crystal concentration, osmotic pressure, and pH value are close to those of CSF, which is basically consistent with the physiological state of CSF circulation and can stabilize the internal environment of brain tissues. It has been shown to be more effective than saline in the treatment of various neurosurgical diseases and is safe and reliable. In a study utilizing a mouse model, it was observed that the choice of irrigation solution employed during neurosurgical procedures could significantly impact the extent of bleeding at the injury site. The study revealed that ACF irrigation was more effective in reducing bleeding as compared to NS (13). Calcium is a crucial component necessary for blood coagulation and vascular smooth muscle contraction, both of which play crucial roles in hemostasis. The prior investigation hypothesized that the presence of Ca^2+^ in ACF could have contributed to the observed reduction in bleeding. In another study, it was reported that ACF irrigation during burr-hole surgery resulted in less damage to CSDH membranes than NS irrigation, leading the authors to speculate that ACF could minimize irritation and promote post-surgical healing (12). These findings suggest that ACF could facilitate hemostasis of the outer hematoma membrane. Moreover, ACF irrigation could help prevent recurrence by washing out inflammatory mediators from the subdural compartment. Excessive inflammation is a primary cause of CSDH, and the overproduction of inflammatory mediators has been linked to hematoma expansion (36). Nevertheless, there are some negative aspects of using ACF, particularly relating to the cost and/or availability of this irrigation fluid worldwide. ACF is not readily available in many neurosurgical centers, but NS most definitely is, as are fluid warmers in most operating suites, and heating NS to body temperature may have a much higher worldwide impact on managing this condition than exploring artificial CSF, particularly in developing countries.

Irrigation at body temperature may be more effective in decreasing postoperative recurrence rates than irrigation at room temperature, partly due to its superior ability to rinse the subdural space of factors that promote hematoma progression. Organic materials’ aqueous solubility doubles with every 20°C increase in temperature, making irrigation fluid at room temperature less effective (18). Moreover, irrigation fluid at room temperature may negatively impact coagulation compared to the fluid at body temperature. Irrigation at body temperature represents a refinement of current surgical practices, easily implemented without any patient-related contraindications or increased risk. Alternatively, irrigation fluid at body temperature may increase hematoma solubility, facilitating evacuation (19).

To our best of knowledge, this is the first meta-analysis investigated the effect of ACF and NS as irrigation fluids, as well as the effect of different irrigation fluid temperatures, on postoperative recurrence rates. The results showed that the ACF group had a lower postoperative recurrence rate than the NS group, possibly due to ACF’s protective effect on cerebral vascular permeability. During surgery, the dura mater may be damaged, and the irrigation fluid may come into contact with the surface of the brain. The severe swelling around the incision after surgery can be alleviated by using ACF, which is similar in composition to CSF and can better maintain the stability of vascular endothelial cells. This can minimize cerebral vascular permeability and cell damage and promote faster hemostasis without interrupting normal coagulation, effectively reducing the recurrence of CSDH. The results also showed that the temperature of the irrigation fluid significantly affected the recurrence rate of CSDH, with the use of body temperature being more effective in reducing postoperative hematoma recurrence than using room temperature. Under room-temperature conditions, the irrigation fluid inhibits the clotting process, leading to non-healing and the recurrence of the hematoma. In contrast, under body temperature conditions, the irrigation fluid increases the solubility of the hematoma, thereby promoting its excretion and absorption. Therefore, it can effectively suppress hematoma formation. In summary, ACF irrigation has a good hemostatic effect and high safety. It is superior to NS in CSDH surgical treatment. Additionally, to minimize the stimulating effect of irrigation fluid temperature on brain tissue, it is recommended to heat the irrigation fluid to body temperature during surgery.

However, this study has some limitations. First, there are few comparative studies on the effects of ACF and NS on CSDH and only four studies on the effect of irrigation fluid temperature on CSDH treatment. Second, there is a lack of research on the effect of ACF temperature on the prevention of CSDH recurrence. Third, the number of included studies in this paper is small, with large differences in quality scores among them. There are also significant differences in ethnicity, time period, and country of origin, with only four RCT studies and seven retrospective case–control studies. Therefore, large-sample, multicenter, well-designed RCTs are still needed to supplement and verify the results of this study. Lastly, the study by Shinbashi et al. (29) was a cohort study using the Japanese patient database from 2010 to 2019. Maybe there is data duplication with other original studies from Japan about different irrigation fluids. But we cannot further identify this possibility. Hence, this is a limitation that cannot be solved.

## Conclusion

Our analysis revealed a significant difference in the choice of irrigation fluid for CSDH surgery. Notably, we found that irrigation with fluid at body temperature demonstrated superiority over irrigation with fluid at room temperature, resulting in fewer instances of recurrence. This straightforward technique is both safe and widely available, providing an opportunity to optimize outcomes for patients with CSDH. Our findings suggest that the use of body-temperature NS or ACF of room temperature during operation should be considered a standard of procedure in CSDH surgery. Nevertheless, whether the different temperature of ACF could be considered a standard of procedure in CSDH surgery still need high-quality RCTs to further identify.

## Data availability statement

The original contributions presented in the study are included in the article/Supplementary material, further inquiries can be directed to the corresponding authors.

## Author contributions

The present study was conceived through the joint efforts of Y-WH, Z-PL and X-SY. Y-WH developed the initial idea. Z-PL and X-SY subsequently devised and refined the search strategy, while Y-WH and X-SY formulated the study design. Y-WH and X-SY contributed to the original draft, and Z-PL was responsible for revising the manuscript. All authors contributed to the article and approved the submitted version.

## Funding

This work was supported by the Project of Mianyang Central Hospital (2021YJ006).

## Conflict of interest

The authors declare that the research was conducted in the absence of any commercial or financial relationships that could be construed as a potential conflict of interest.

## Publisher’s note

All claims expressed in this article are solely those of the authors and do not necessarily represent those of their affiliated organizations, or those of the publisher, the editors and the reviewers. Any product that may be evaluated in this article, or claim that may be made by its manufacturer, is not guaranteed or endorsed by the publisher.

## Footnotes

^1^https://www.crd.york.ac.uk/PROSPERO/
